# Ion-dependent slow protein release from *in vivo* disintegrating micro-granules

**DOI:** 10.1080/10717544.2021.1998249

**Published:** 2021-11-08

**Authors:** Patricia Álamo, Eloi Parladé, Hèctor López-Laguna, Eric Voltà-Durán, Ugutz Unzueta, Esther Vazquez, Ramon Mangues, Antonio Villaverde

**Affiliations:** a Biomedical Research Institute Sant Pau (IIB Sant Pau), Barcelona, Spain; b Josep Carreras Leukaemia Research Institute (IJC Campus Sant Pau), Barcelona, Spain; c CIBER de Bioingeniería, Biomateriales y Nanomedicina (CIBER-BBN), Madrid, Spain; d Institut de Biotecnologia i de Biomedicina, Universitat Autònoma de Barcelona, Bellaterra, Spain; e Departament de Genètica i de Microbiologia, Universitat Autònoma de Barcelona, Bellaterra, Spain

**Keywords:** Protein materials, microparticles, protein depots, self-disintegrating materials, tumor targeting

## Abstract

Through the controlled addition of divalent cations, polyhistidine-tagged proteins can be clustered in form of chemically pure and mechanically stable micron-scale particles. Under physiological conditions, these materials act as self-disintegrating protein depots for the progressive release of the forming polypeptide, with potential applications in protein drug delivery, diagnosis, or theragnosis. Here we have explored the *in vivo* disintegration pattern of a set of such depots, upon subcutaneous administration in mice. These microparticles were fabricated with cationic forms of either Zn, Ca, Mg, or Mn, which abound in the mammalian body. By using a CXCR4-targeted fluorescent protein as a reporter building block we categorized those cations regarding their ability to persist in the administration site and to sustain a slow release of functional protein. Ca^2+^ and specially Zn^2+^ have been observed as particularly good promoters of time-prolonged protein leakage. The released polypeptides result is available for selective molecular interactions, such as specific fluorescent labeling of tumor tissues, in which the protein reaches nearly steady levels.

## Introduction

Many pharmacological treatments for chronic diseases, such as cancer would benefit from a time-prolonged drug supply aiming at reaching constant or nearly constant levels at the site of action (Natarajan et al., 2014; Koshy et al., 2018; Cross et al., 2019). This is in contrast with the common therapeutic protocols that are based on repetitive drug administrations, frequently given with a few-day time intervals, resulting in oscillating drug concentrations and irregular therapeutic impact (Wen et al., 2015; Zou et al., 2020). Approaching steady drug concentrations in blood is expected to minimize side effects in off-target tissues and to support a potent therapeutic action (Rosen & Abribat, 2005; Wen et al., 2015; Li et al., 2019; Zou et al., 2020), while non-repetitive administrations would reduce the exploitation of sanitary resources (Pareek et al., 2019). Diverse strategies for sustained drug delivery are under exploration, mainly based on porous materials, hydrogels, matrices, or other types of macroscopic, microscale, or nanoscale containers that hold the drug for its progressive leakage (Gilmore et al., 2016; Li & Mooney, 2016; Ali & Ahmed, 2018; Ghalei et al., 2018; Koshy et al., 2018; Wu et al., 2018; Meng et al., 2019). Such methodologies involve a non-drug-containing material that increases the complexity of the system and the fabrication process, often imposing chemical constraints and toxicological concerns (Sharma et al., 2012; Palombo et al., 2014; Aragao-Santiago et al., 2016; Shen et al., 2017). Recently, self-contained, self-disintegrating protein material in form of microparticles have been developed (Chen et al., 2020; Sánchez et al., 2020), suited for a slow protein release *in vivo* (Sánchez et al., 2020; Serna et al., 2020; López-Laguna et al., 2021). These artificial structures mimic the molecular organization and functionality of the secretory granules from the mammalian endocrine system, which contain and release peptidic hormones to the bloodstream (Maji et al., 2009; Mankar et al., 2011; Jacob et al., 2016; Jacob et al., 2019). Such artificial material is based on pure preparations of a single polypeptide species with a fused histidine-rich peptide, and that is clustered as granules around the microscale, by the external addition of Zn^2+^. The divalent cation generates cross-molecular interactions between histidine-rich domains of adjacent polypeptide chains, which remain attached by such interactions in form of a mechanically stable protein network (López-Laguna et al., 2020). Upon *in vitro* incubation under physiological conditions or by subcutaneous administration *in vivo*, the protein granules spontaneously disintegrate, probably by progressive chelation of the clustering ions. This fact allows a slow leakage of the protein building blocks, in a functional form, ready for interactivity or any other biological activity. Divalent non-toxic cations other than Zn^2+^ are, in principle, potentially suited for protein clustering (López-Laguna et al., 2020). By exploring these alternatives, a diversity of related materials could be generated whose properties, regarding the kinetics of protein release, functionality and bioavailability, are presumed to be differential.

## Materials and methods

### Manufacture of secretory granules and release of soluble protein

**Figure 1.**
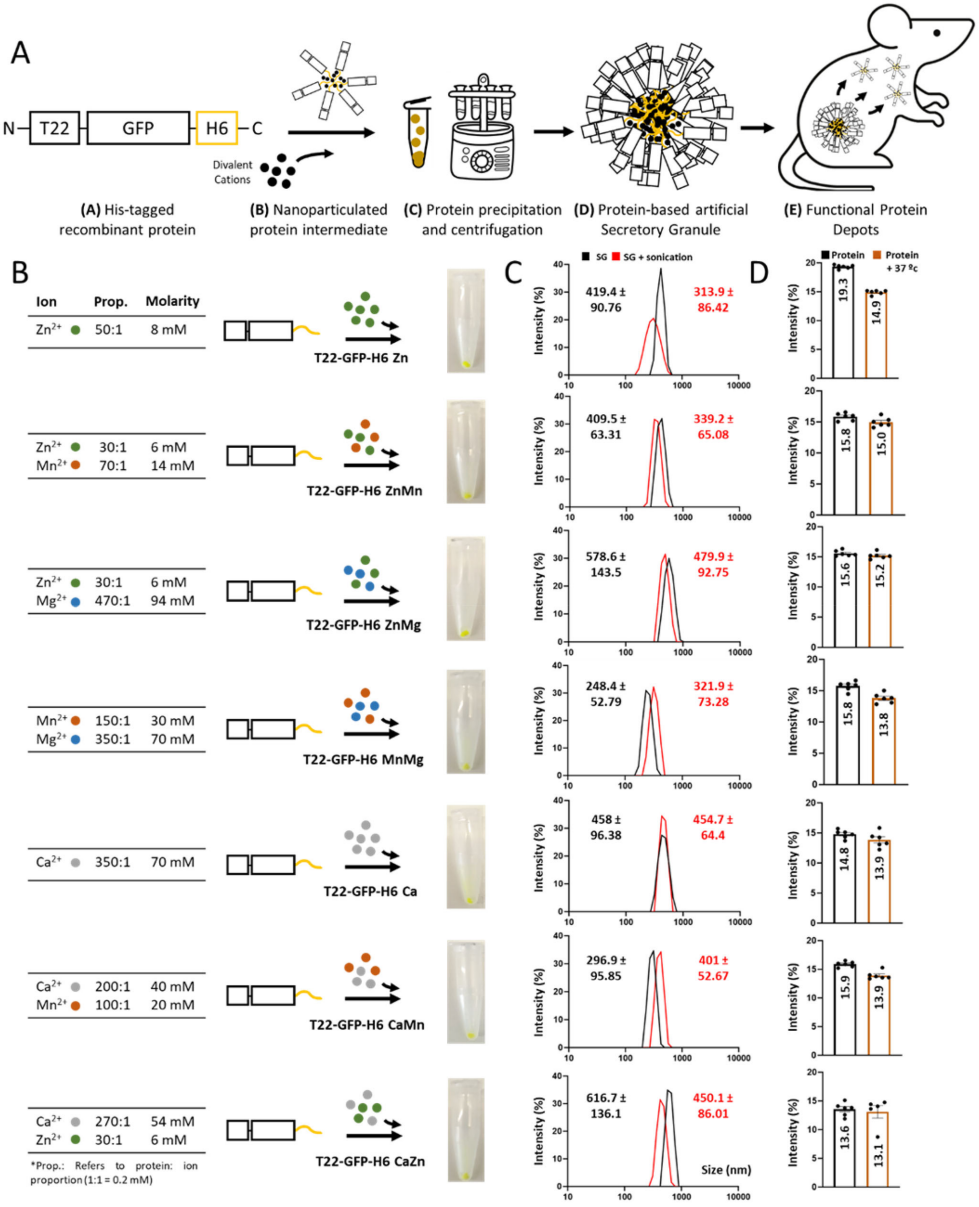
Formulation and physicochemical characterization of secretory granules using different combinations of divalent cations. (A) Schematic representation of the manufacturing process. Architectonically stable protein stages are depicted down below each picture. More details about the organization of final and intermediate materials can be found elsewhere (López-Laguna et al., 2021). (B) Methodological procedure of secretory granules displaying different types of divalent cations and concentrations. Pictures of the resultant pellets are also displayed. (C) Size determination (in nm) by DLS after the preparation of secretory granules (black). Size determination upon sonication (1 min, 10% amplitude, and 0.5 s on/off) to address mechanical stability (red). SG refers to Secretory Granule. Displayed values correspond to peak sizes. (D) Size determination (in nm) by DLS of released soluble protein from secretory granules (black). Size determination upon thermal exposure (37 °C for 24 h) to address thermal stability (brown). All measurements were performed in sextuplicate, and data was represented as mean ± SEM (standard error of the mean).

Pure soluble T22-GFP-H6 [Figure 1(A), fully described elsewhere (Rueda et al., 2015)], was aliquoted in 250 µL at 2 mg/mL in the storage buffer (166 mM NaCO3H + 333 mM NaCl). Protein precipitation as secretory granules was induced by the direct addition of divalent cations to protein solutions. Different cation: protein molar proportions (and therefore working ion concentrations) were used for the different types of granules, depending on the crosslinking ions. The molar protein amount refers to the molar amount of histidine residues in the overhanging H6 tag (that is, the raw protein molar value x 6). For the construction of Zn-based depots we used a molar ratio 50:1 (at 10 mM Zn^2+^); for ZnMn-based depots, the ratios were 30:1 (Zn^2+^) and 70:1 (Mn^2+^) (at 6 mM Zn^2+^ and 14 mM Mn^2+^); for ZnMg-based depots, 30:1 Zn^2+^ and 470:1 Mg^2+^ (at 6 mM Zn^2+^ and 94 mM Mg^2+^); for MnMg depots, 150:1 Mn^2+^ and 350:1 Mg^2+^ (at 30 mM Mn^2+^ and 70 mM Mg^2+^); for Ca-based depots, the ratio was 350:1 (using 70 mM Ca^2+^); for CaMn-based depots, ratios were 200:1 Ca^2+^ and 100:1 Mn^2+^ (at 40 mM Ca^2+^ and 20 mM Mn^2+^); for CaZn-based depots, 270:1 Ca^2+^ and 30:1 Zn^2+^ (at 54 mM Ca^2+^ and 6 mM Zn^2+^) (see Figure 1(B)). Proportion 1:1 refers to 0.2 mM of both protein and ion. Samples were then incubated at room temperature for 10 min and subsequently centrifuged at 10,000 g and room temperature for 10 min to separate the soluble from the insoluble protein fractions. The insoluble fraction (namely secretory granules) was collected and stored at −80 °C for further use, and the soluble fraction was quantified by Bradford's assay (Maniatis et al., 1989) to determine the amount of precipitated protein in mg. Bacterial inclusion bodies formed by T22-GFP-H6 were prepared by standard bacterial production procedures (Cespedes et al., 2020).

The release of soluble protein from secretory granules was triggered *in vitro* by the direct addition of 250 µL of storage buffer (166 mM NaCO_3_H +333 mM NaCl) into the thawed insoluble fraction (namely secretory granules). Samples were properly mixed using a pipette for several minutes and centrifuged at 10,000 g and room temperature for 10 min to collect the soluble fraction (namely released soluble protein).

### Size determination of secretory granules and the released soluble protein

The hydrodynamic diameter (in nm) of secretory granules and soluble protein released *in vitro* was determined by dynamic light scattering (DLS) at 25 °C (633 nm), run duration (0.839 s), the number of runs 15, using the forward scatter (for secretory granules) and backscatter (for released soluble protein) detectors, in a Zetasizer NanoZS (Malvern Instruments Limited) using ZEN2112 3 mm quartz batch cuvettes.

### *In vivo* kinetic biodistribution of fluorescent material by subcutaneously implanted granules

*In vivo* experiments were approved by the Animal Ethics Committee at Hospital de la Santa Creu i Sant Pau (procedure 115_9721) and performed according to European Council directives. To generate the CXCR4^+^ SW1417 CRC cancer model, four-week-old female mice of the Swiss nude strain, in the 18–20 g body weight range (Charles River, L-Abreslle, France) and maintained in pathogen-free conditions, were used. We injected subcutaneously in the mouse flank (*n*=) 5 × 10^6^ CXCR4^+^ SW1417 CXCR4^+^ human CRC cells, resuspended in 150 μL of media. When tumors reached approximately a 120–200 mm^3^ volume, animals were randomly allocated to the different groups and administered in the subcutis of the mouse lumbar region, at the side contralateral to the tumor site, with a pellet of 1 mg of T22-GFP-H6 granules suspended in a 150 μL PBS buffer. Inclusion bodies formed by a recombinant T22-GFP-H6 and purified from bacteria or PBS buffer were injected as controls.

Ten minutes, 5, 10, 24, 48, 120, or 600 h after the administration, mice were registered *in vivo*. The mouse was euthanized, and the kidney, liver, tumor, and tissues surrounding the injection point were resected. Following, we *ex vivo* registered the intensity of the fluorescence emitted by the protein released by the protein depots and biodistributed to the tumor and non-tumor organs and also the fluorescence remaining the injection site, using the IVIS^®^ 200‐Spectrum (PerkinElmer, Waltham, MA, USA). The fluorescent signal was digitalized, displayed as pseudocolor overlay, and expressed as radiant efficiency. All experimental data points were corrected by subtracting the mean average radiant efficiency of each respective tissue from mice injected with PBS. Preliminary screening was conducted with one mouse per condition while the final *ex vivo* assay was, at least, performed in duplicate.

### Statistical analyses

All statistical analyses and data representation were performed in Graph Pad Prism (v8.0.2).

## Results and discussion

To refine the design of such disintegrable protein drug depots with potential for therapeutic applications we have explored here the use of different divalent cations for the construction of secretory granules of a reporter fluorescent protein (T22-GFP-H6, Figure 1(A)) produced and purified from recombinant bacteria. This has been done to comparatively evaluate how the forming building blocks leak from the depot at the subcutaneous injection site. Several divalent cations, namely those of Zn, Ca, Mn, and Mg, found at relatively high concentrations in living beings, were selected to prevent potential toxicity issues linked to the molecular glue. These physiological linkers were applied to generate, *in vitro*, secretory granules of the modular protein T22-GFP-H6, through a process in which homomeric nanoparticles are intermediates (López-Laguna et al., 2021, Figure 1(A)). This polypeptide contains an enhanced GFP as a core protein for straightforward monitoring of biodistribution, flanked by a C-terminal hexahistidine tail (H6) and an N-terminal cationic peptide, T22. T22 is a ligand of the tumoral marker CXCR4 (Tamamura et al., 1993, 1998a, 1998b), that in form of a recombinant fusion version promotes a highly selective binding and penetration into CXCR4^+^ cells. Also, it’s *in vivo* accumulation in tumoral tissues is detectable through fluorescence (Cespedes et al., 2018; Falgas et al., 2020a, 2020b; Pallares et al., 2020). The combination of T22 and GFP in a single modular polypeptide is then suitable to estimate not only the protein leakage from the injection site but also its final fate when using animal models of CXCR4^+^ cancers. Also, T22-GFP-H6, in contrast to other previously tested proteins (López-Laguna et al., 2021), is well-aggregated *in vitro* by Zn^2+^ as mechanically stable materials (López-Laguna et al., 2021). In physiological buffer, these clusters release oligomers of the protein (Figure 1(A)) sizing around 13–14 nm (López-Laguna et al., 2021), a size very similar to that reached by the soluble protein version upon a spontaneous self-assembling process (around 12 nm) (Lopez-Laguna et al., 2019).

In this regard, seven versions of T22-GFP-H6 (Figure 1(B)), clustered as mechanically stable micron-scale protein granules (Figure 1(C)) were generated through alternative cations or cation mixtures for subcutaneous administration, a route that allows entry of any leaked protein into the bloodstream but also a local permanence of the remaining material as a depot (Unzueta et al., 2018). The elements selected for clustering were Zn and Ca, alone or as mixtures with Mg and Mn, namely ZnMn, ZnMg, MnMg, CaMn, and CaZn (Figure 1(B)). These cations were selected for their regular presence and relative abundance in living beings (Knape et al., 2017; Pilchova et al., 2017; Al Alawi et al., 2018; Li & Yang, 2018; Pazirandeh et al., 2020; Santos et al., 2020), thus skipping toxicities potentially linked to rarer oligoelements. These ions also cover a wide range in the Irving-Williams (Milicevic et al., 2011) series, which predicts differential stability of the complexes formed by those cations through coordination with histidine residues. In addition, we tested growing cation: protein molar ratios to further expand the range of protein outflow (Figure 1(B)). By using these cations and mixtures we looked for the generation of granules with distinct leakage properties and therefore, different therapeutic potential and applicability in living systems. All the generated granules, with sizes ranging between 300 and 600 nm, resulted mechanically stable as they resisted sonication with only slight size modifications (Figure 1(C)). Also, they exclusively leak, *in vitro*, nanoparticles (but not monomers) of around 13–15 nm that are also structurally stable upon *in vitro* incubation in the physiological buffer for at least 24 h at 37 °C (Figure 1(D)). This observation suggested that the leaked material could be also stable in physiological fluids.

**Figure 2.**
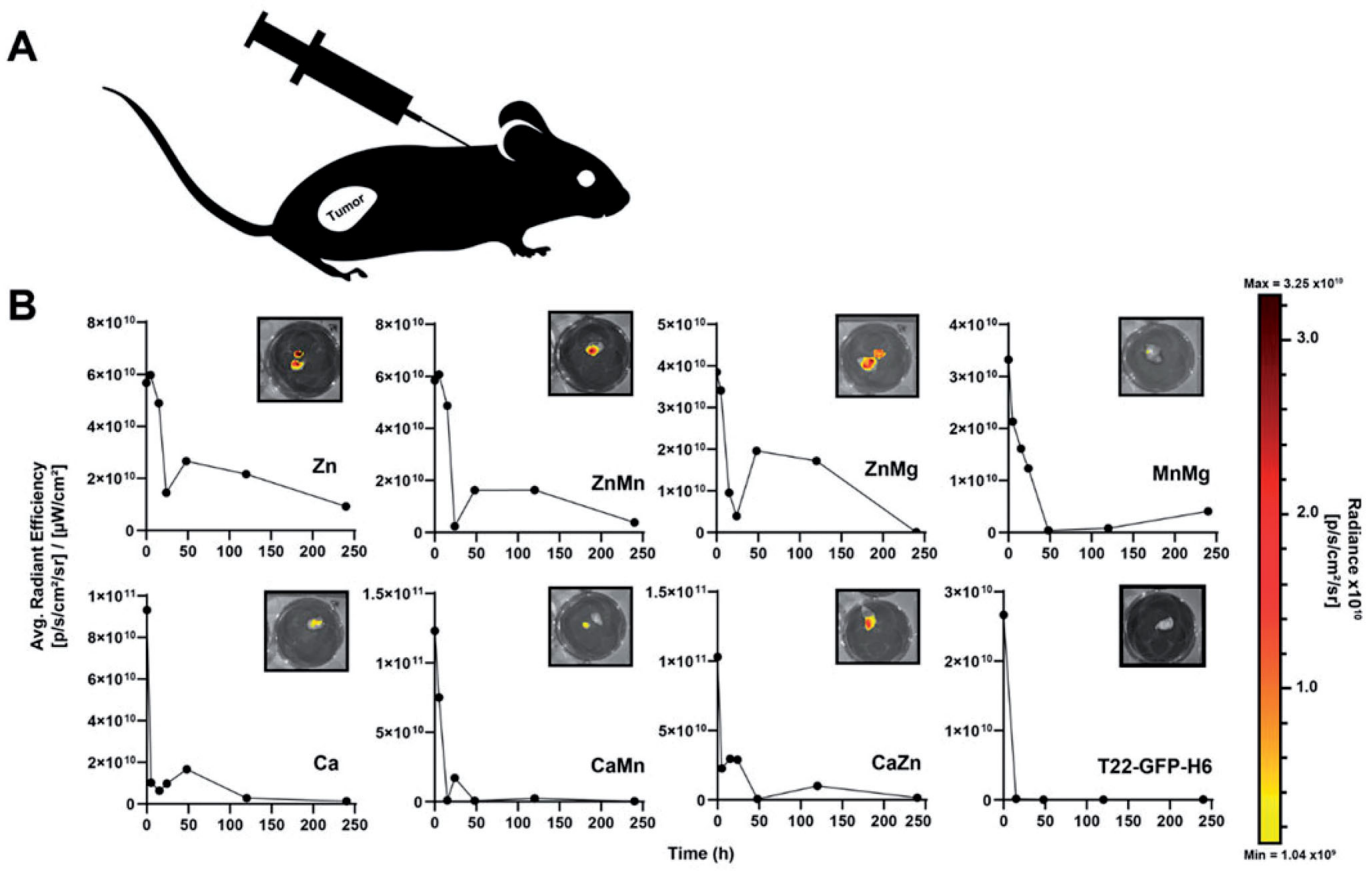
Preliminary screening of protein leakage from the secretory granules. (A) Representation of the injection site, in a contralateral area regarding the tumor in the SW1417 colorectal cancer model. (B) Temporal loss of protein material at the injection point monitored *in vivo* through the emitted fluorescence. In the insets, IVIS images were recorded at 5 days (120 h) post-administration. The scale color bar reflects the radiance expressed as (p/s/cm^2^/sr).

For a fast initial screening, 1 mg of each sample was subcutaneously injected in the CXCR4-expressing SW1417 human colorectal cancer mouse model, as a single dose, in a contralateral remote area regarding the tumor location (Figure 2(A)). The material remaining at the injection point was monitored through fluorescence as an indication of protein loss, during 10 days upon administration. As observed, an inclusion body version of the protein that also releases fluorescent protein material (Unzueta et al., 2018) acting as naturally produced secretory amyloids (Unzueta et al., 2018; Cespedes et al., 2020), was unable to stay at the injection point for long time periods post-administration (Figure 2(B)). In contrast, and in general terms, all the clustered materials were observed as valid depots from which the forming protein was progressively released in comparison to the control, soluble non-clustered protein (Figure 2(B)). However, clear dissimilarities in the protein release rate were also observed. While with some differences, Zn- and Ca-containing materials tended to lose fluorescence more progressively. In contrast, materials in which Mn and Mg participated, including the MnMg combination, showed a tendency to release the protein in a faster way. The MnMg combination resulted in fact in the material disintegrating more rapidly (Figure 2(B)).

**Figure 3.**
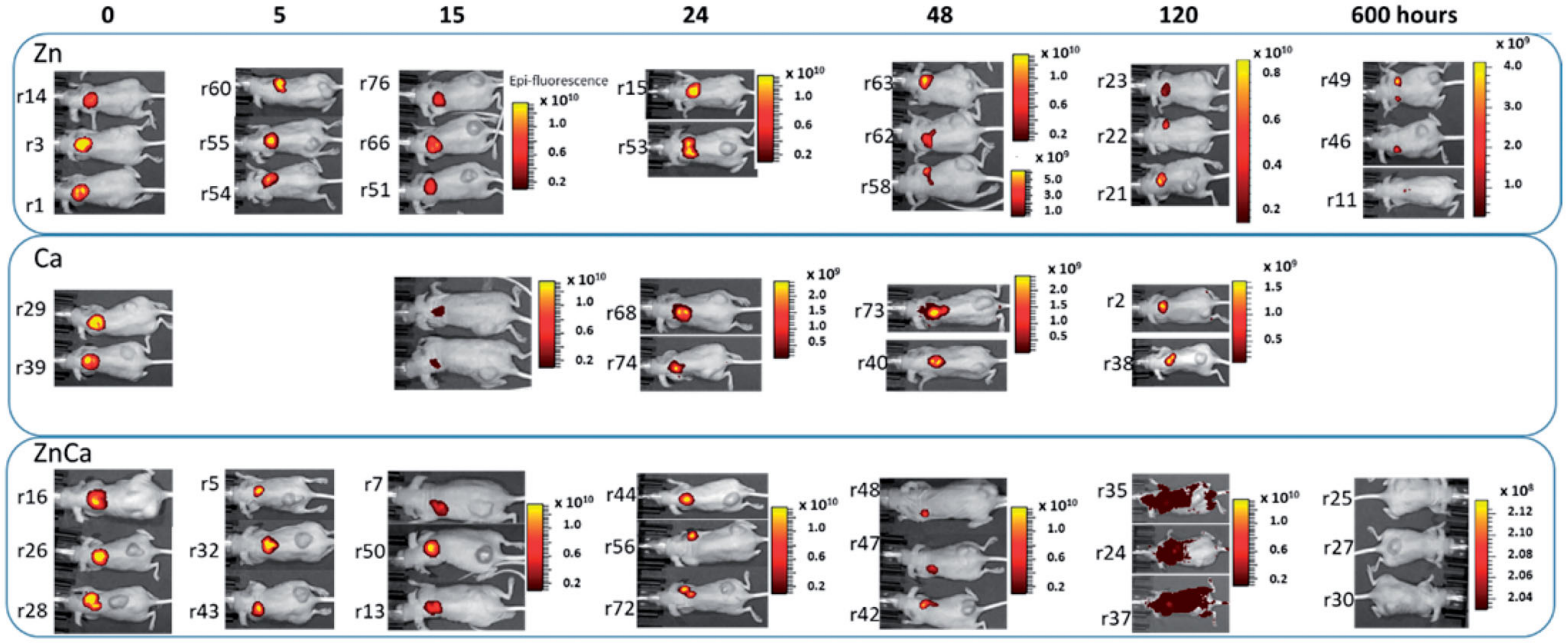
Whole animal IVIS imaging in which the administered material confers fluorescence at the injection point. The administered protein was clustered with divalent cations of either Zn, Ca, or a CaZn mixture as indicated in Figure 1. Numbers at left indicate the particular animal from which the final *ex vivo* reading was recorded (see Supplementary Figure 1). The scale color bar reflects the radiance expressed as (p/s/cm^2^/sr).

Although these data resulted only just from a preliminary screening, the rather consistent negative impact of Mn^2+^ and Mg^2+^ on the permanence of the material at the injection site made us presume that the materials resulting from those ions are more unstable than their counterparts. Then, looking for a time-prolonged release, we took a deeper exploration of the depot potential of Zn- and Ca-based materials. Granules formed with the assistance of either Zn, Ca or a CaZn combination of divalent cations was administered again in the mouse model of human, CXCR4^+^ SW1417 colorectal cancer, in a larger number of animals. The administration was done in the contralateral area relative to subcutaneous tumors (Figure 2(A)). The loss of fluorescence at the injection point was monitored during relatively long periods of time in whole animals, spanning from the immediate administration to 10 days post-injection (Figure 3). Again, the immediate visualization of fluorescence at the injection point was indicative of an extended permanence of the material in the subcutaneous depot, being Zn^2+^ the clustering ion supporting a slower protein release. However, the whole animal imaging was not precise enough for a fine quantification, and for observing the fate of the released materials. Expectedly, if our starting hypothesis was correct, the protein resulting from the depot disintegration should generate steady fluorescence levels in the CXCR4^+^ tumor, because of the presence of T22 as a targeting agent (Figure 1(A)).

**Figure 4.**
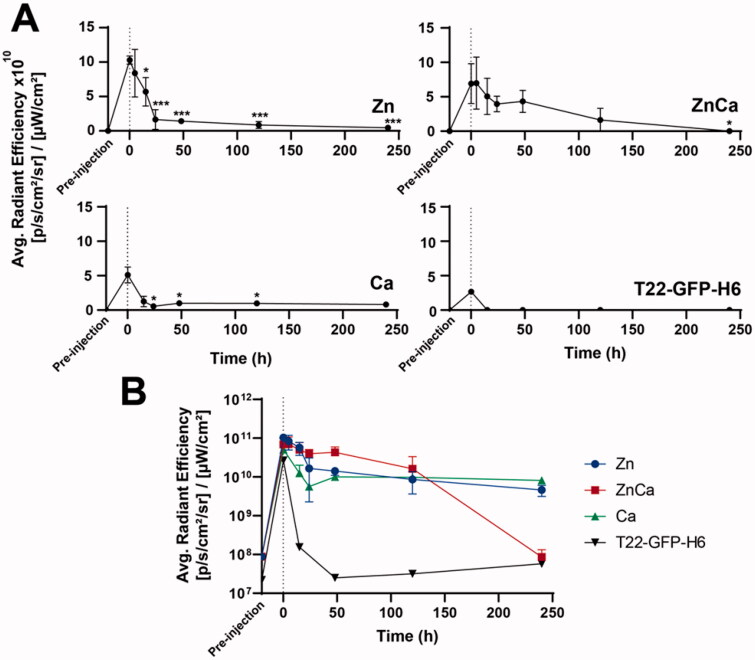
Kinetics of protein depot leakage upon *ex vivo* examination, no background subtraction. (A) Individual kinetics of protein leakage upon subcutaneous administration of either Zn-, Ca-, or CaZn-based granules. Soluble T22-GFP-H6 (in form of nanoparticles) was injected as a control. (B) A comparative plotting of the above data. Differences with data at time 0; *p ≤ .05, ***p ≤ .001.

For that, at the times indicated in Figure 3, animals were euthanized for the *ex vivo* determination of GFP fluorescence in the depots and in the target tumor (Supplementary Figure 1). By the analysis of these *ex vivo* data, the release kinetics of each type of depot was determined. As observed (Figure 4(A,B)), in contrast to inclusion body T22-GFP-H6 that dropped immediately from the injection site, the artificial submicron granules generated with the assistance of ions allowed a sustained protein release, keeping significant amounts of the starting material at the injection point 10 days upon administration (Figure 4, Supplementary Figure 1). Ca-based materials tended to be less supportive of protein permanence than Zn-based counterparts. Then, at a few hours upon administration, an important leakage of the protein was observed followed by a smoother release phase (Figure 4(A,B)). In the hybrid CaZn-clustered material, Ca appeared as a negative regulator of protein retention, favoring release. This is an interesting observation as it appears that this cation could be used as a modulator to adjust the leakage rate if designing very precise kinetics is envisaged.

**Figure 5.**
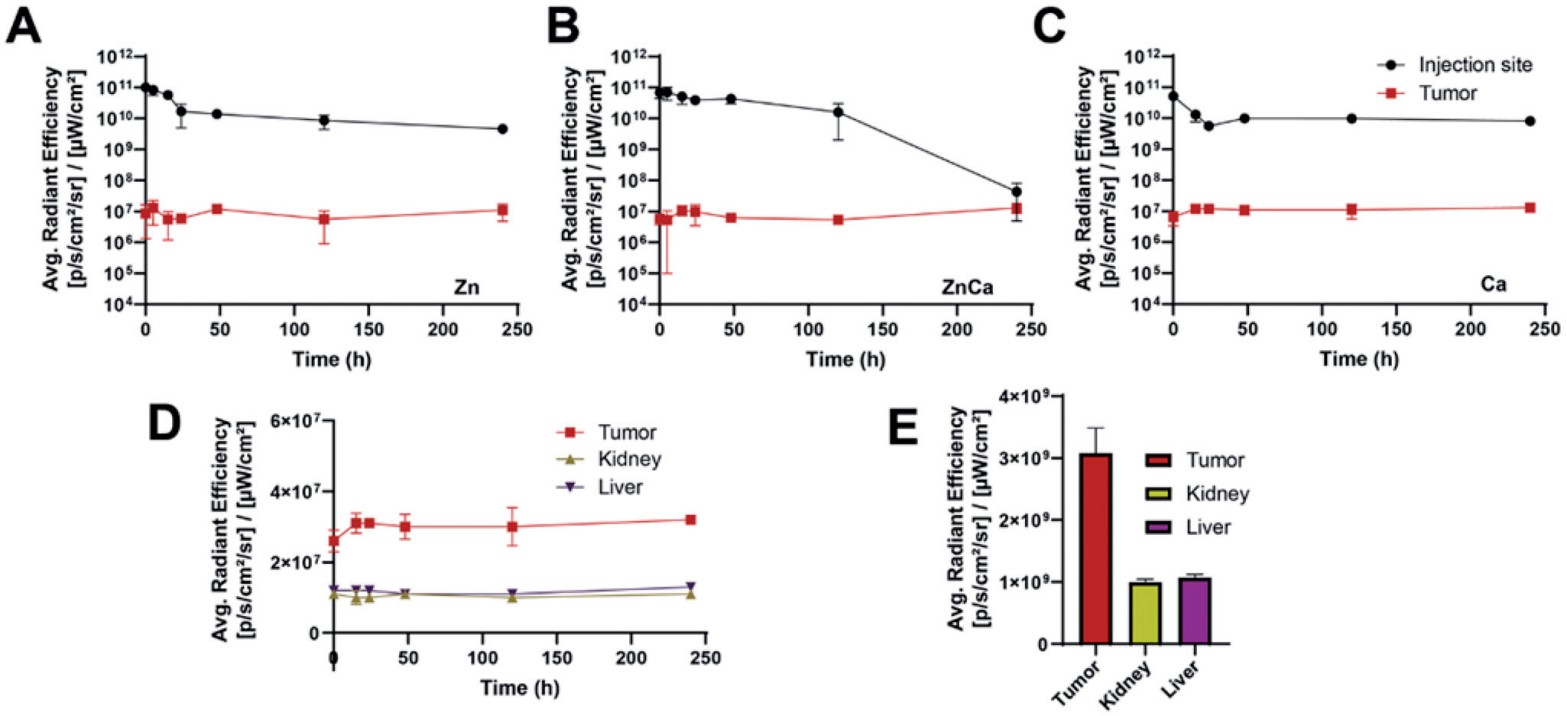
Protein levels at the CXCR4^+^ target tumor. Comparative plotting of fluorescence levels at both the injection site and in tumor upon *ex vivo* analysis, for Zn-based (A), Ca-based (B), or CaZn-based protein depots (C). In Ca-based depots, fluorescence levels in the tumor are plotted together with those in the liver or kidney (D), and the global area under those curves in the monitored time interval is represented for a simple comparison (E).

Noting this long permanence of the aggregated protein at the injection site, it would be interesting to know the fate of the released fraction and especially, if the granule-based protein delivery might ensure a steady level of the removed protein at the target site. Such target tissue is, in this system, the CXCR4^+^ tumor toward which the T22 peptide is directed. The *ex vivo* analysis of the fluorescence in the tumor revealed steady levels of fluorescence along the monitored time (Figure 5(A); Supplementary Figure 1). The intensity of the fluorescence in the tumor was between 200 and 300 times lower than those observed at the injection site. An exception was the animals treated with CaZn granules in which the fluorescence at the depot dropped dramatically 10 days upon injection. Interestingly, the observed sets of fluorescence values were rather similar in the three types of tested materials (Figure 5(A)). The protein released from Ca-based granules was particularly steady, as the fluorescence values in the tumor were very constant from a few hours upon injection on, compared to the slightly fluctuating values in the two alternative systems. Because of such stability, we selected the Ca-based model to evaluate, in parallel, the occurrence of GFP fluorescence in two main off-targeted organs, namely the kidney and liver. In this regard, the fluorescence in the tumor was three times higher than that observed in these organs, as evaluated visually by plain kinetic curves (Figure 5(D)), or numerically through the area below the curve (Figure 5(E)). Therefore, T22-GFP-H6 was not only released from the subcutaneous depots during at least 10 days, but the leaked protein also reached steady levels in a target tissue, which are significantly higher than background levels in the kidney and in the liver.

Altogether, these data indicate a promising pattern of the slow disintegration of subcutaneously administered secretory granules, fabricated *in vitro* by the use of divalent cations as protein clustering agents. Importantly, the offered data indicates that the selection of the involved ion determines the strength of the protein-protein contacts and therefore, the rate of protein release during *in vivo* disintegration of the material (Figures 1–3). Importantly, the protein is released from these depots for at least 10 days (Figures 2–4), and it reaches the target organ in a functional form, ensuring steady local levels during the whole experimental time (Figure 5).

We had previously demonstrated that bacterial inclusion bodies, a type of non-toxic amyloids found in recombinant bacteria (de Marco et al., 2019), can release the forming protein in a functional form, upon local (Cespedes et al., 2016; Pesarrodona et al., 2019) or remote (Unzueta et al., 2018; Cespedes et al., 2020) administration to animals, for a therapeutic effect in breast and colorectal cancer models (Pesarrodona et al., 2019; Cespedes et al., 2020). However, inclusion bodies are highly heterogeneous materials regarding composition, and they trap, during the aggregation of the recombinant protein in the bacterial cytoplasm and the further purification process, numerous bacterial molecules including proteins, nucleic acids, and cell wall components (de Marco et al., 2019). This fact might pose regulatory limitations to the clinical applicability of this material, whose natural formation in the cells cannot be controlled externally and the disintegration process appears to be too fast for a true time-prolonged delivery system (Figure 2). The materials presented here, that are mimetics of those natural inclusion bodies (Sánchez et al., 2020; Sanchez et al., 2021), undergo a chemically controlled fabrication process from a purified protein that is clustered, in a controlled process, by physiological concentrations of divalent cations present in the body. Importantly, the results reported in the present study indicate that the release kinetics from these materials, upon subcutaneous administration, is time-sustained enough to represent a promising candidate for a slow drug delivery system. Importantly, the disintegration process can be regulated in the upstream section of the fabrication by the proper selection of the clustering ion. Among those tested (Figure 1(B)), Zn^2+^ and Ca^2+^ resulted in especially promising as they favor the retention of the polypeptides in the depot and extend their release into the body (Figure 5). The released protein is fluorescent and fully targeted to the tumor in an animal model of human colorectal cancer, accumulating in such a fluorescent form in tumor tissues (Figure 5, Supplementary Figure 1). This fact indicates that apart from mere therapeutic uses, secretory granules might be also used in theragnosis as the time-prolonged release of tumor-targeted protein markers from remote repositories allows a precise visualization of tumor foci. Other strategies have been already developed that use metal coordination to stabilize different categories of nanoparticles for drug delivery (He et al., 2019, 2020), taking advantage of the coordination capacities of Zn and other metals (López-Laguna et al., 2020). On the other hand, metal coordination with polyhistidine stretches has allowed the controlled oligomerization of peptide and protein materials *in vitro*, for the construction of nanostructured immunogens (Manuel-Cabrera et al., 2016), nanotubes (Yewdall et al., 2018), and nanowires (Zhang et al., 2012), among many others (López-Laguna et al., 2020). The exploitation and adaptation of metal and non-metal divalent cations to construct self-disintegrating secretory granules opens a spectrum of possibilities in therapy and diagnosis that could be hardly reached by alternative systems, offering in addition an important extent of versatility regarding the used ion and its capability to retain and leak the protein building blocks.

Altogether, these data and concepts point out synthetic protein granules composed of ion-clustered his-tagged proteins as a regulatable secretory platform for clinical applications that being self-disintegrating, does not need, compared to alternative approaches (Petlin et al., 2017; Ali & Ahmed, 2018; Saghazadeh et al., 2018; Stewart et al., 2018; Safdar et al., 2019), any scaffold material for drug hosting.

## Data Availability

The data that support the findings of this study are openly available in DDD (UAB) at https://doi.org/10.5565/ddd.uab.cat/249709 or https://ddd.uab.cat/record/249709.
